# Evaluation of an automated seed loader for seed calibration in prostate brachytherapy

**DOI:** 10.1120/jacmp.v7i1.2114

**Published:** 2006-02-21

**Authors:** Shuying Wan, Chandra P. Joshi, Greg Carnes, L. John Schreiner

**Affiliations:** ^1^ Cancer Centre of Southeastern Ontario 25 King Street West Kingston Ontario K7L 5P9 Canada; ^2^ Queen's University 25 King Street West Kingston Ontario K7L 5P9 Canada

**Keywords:** air kerma strength, bead variability, accuracy, standard deviation, reproducibility

## Abstract

Automated seed loaders for permanent prostate implants are now commercially available. Besides improved radiation safety, these systems offer seed assay capability and ease of needle loading, making preplanned as well as intra‐operative implant procedures more time‐efficient. The Isoloader (Mentor Corp., CA) uses individual $I^{125}$ seeds (SL‐125 ProstaSeed) loaded in up to 199 chambers inside a shielded cartridge. The unit performs seed counting and calibration using a built‐in solid‐state detector. In order to evaluate the reproducibility and accuracy of the calibration process, two test cartridges were measured with the Isoloader itself and compared with a well‐type ionization chamber (HDR‐1000Plus, Standard Imaging).

The air kerma strength measurements for all seeds using the Isoloader had a standard deviation of about 2.7%. For the eight seeds assayed more intensively using both the Isoloader and well chamber, the standard deviations of the measurements for each seed were in the range of 0.8% to 2.8% and 0.6% to 1.3%, respectively. The variation in the Isoloader calibration is attributed to small detector solid angle and bead geometry within seed capsules (verified by radiographs). The reproducibility of the air kerma strength measured by the Isoloader was comparable to that from the well chamber and was clinically acceptable. Seed strength measured with the Isoloader was on average 1%~2% larger than that measured with the well chamber, indicating that the accuracy of the Isoloader was clinically acceptable.

PACS numbers: 87.53.Jw, 87.53.Xd, 87.56.Fc

## I. INTRODUCTION

Trans‐rectal ultrasound‐guided permanent implantation of radioactive $I^{125}$ and ${\text{Pd}}^{103}$ seeds is an important treatment option for early stage prostate cancer.^(1)^ Such prostate brachytherapy has become increasingly attractive to patients because it is a single‐day outpatient procedure with good treatment efficacy and with reduction in erectile dysfunction.^(2)^ In addition, the development of new technology has made overall planning and treatment execution more accurate and less resource demanding.

Recently automated seed loaders for permanent implants, such as the Isoloader (Mentor Corp., CA) and the FIRST System (Nucletron, the Netherlands), have become commercially available. Besides improved radiation safety, these systems offer seed assay capability and ease of needle loading, making preplanned as well as intra‐operative implant procedures more time‐efficient.^(3)^ At the Cancer Centre of Southeastern Ontario, we use a Mentor Isoloader for needle loadings based on a preimplant plan. The Isoloader uses individual $I^{125}$ seeds (SL‐125 ProstaSeed, Mills Biopharmaceuticals, Okalahoma City, OK) loaded in a ready‐to‐use device called the IsoCartridge. The Isoloader unit performs seed counting and calibration using a built‐in solid‐state detector. This is a new technology, so there are currently sparse guidelines available for automated seed loaders and their seed calibration procedure. The increasing use of these automated systems warrants an evaluation of system performance. This paper presents the results of the seed assay capabilities of the Mentor Isoloader.

The AAPM Task Group 40 (TG40)^(4)^ recommends that brachytherapy seeds should have air kerma strength calibrations with direct or secondary traceability to the National Institute of Standards and Technology (NIST) or an AAPM‐Accredited Dosimetric Calibration Laboratory (ADCL). It also recommends that, for batches with a large number of seeds, a random sample containing at least 10% seeds from the batch should be calibrated. This is the practice of the institutions relying on well‐type ionization chambers for seed calibration, where the well chambers are calibrated at NIST/ADCL.

Since the process of seed calibration with a well chamber is quite tedious and time‐consuming, a quick seed assay using an automated seed loader is attractive. However, instead of being directly calibrated at NIST/ADCL, an Isoloader is initially calibrated using a seed with direct traceability. To ensure the stability of the detector, we requested that the supplier biannually provide for us a seed with secondary traceability and measure it with the Isoloader. TG40 considers the use of “remote traceability” (i.e., calibration by the supplier) acceptable for new calibrators and new seeds; therefore, such calibrations can be considered reliable. However, long‐term users may require more robust guidelines for using automated seed loaders for seed calibrations.

In this paper we report results of a study to assess the quick seed assay facility provided by the Isoloader. The reproducibility and accuracy of the internal Isoloader calibration process were evaluated by measurements with the internal system and with a well‐type ionization chamber on two test IsoCartridges.

## II. METHODS AND MATERIALS

Two batches of $I^{125}$ seeds were acquired from Mentor. All seeds from both batches were measured 7 to 10 times and a few seeds 100 to 200 times using the Isoloader. Ten seeds were then ejected from the cartridges and measured extensively using a well‐type ionization chamber. The details are described here.

### A. $I^{125}$ seed and IsoCartridge

**Figure 1:**
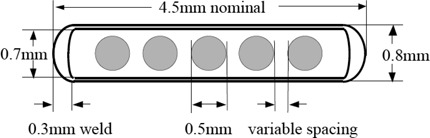
Model SL‐125 ProstaSeed encapsulation design.^(6)^ The five silver beads are coated with radioactive $I^{125}$ on the surface.

An $I^{125}$ seed (SL‐125 ProstaSeed) contains five silver beads packed in a titanium cylindrical capsule with a nominal length of 4.5 mm and diameter of 0.8 mm (see Fig. 1). The capsule wall is 0.05 mm thick, and the laser‐weld ends are 0.3 mm thick (nominally). Each silver bead measures 0.5 mm in diameter and is coated with radioactive $I^{125}$ on the surface.^(^ ^5^ ^–^ ^7^ ^)^ Because of the internal spacing, the beads are movable within the capsule, resulting in a potential changeable geometrical configuration of bead arrangement depending on seed orientation and motion. This inherent variability in the activity distribution within each seed is referred as “bead variability” in this paper.

The $I^{125}$ seeds are preloaded by the manufacturer in up to 199 chambers inside a sterilized and shielded IsoCartridge. Each chamber can accommodate a maximum of 10 mm length of material, which can be filled either by one seed and one short spacer (5.5 mm long) or by one long spacer (10 mm long) only. An IsoCartridge for a typical clinical implant at our center contains 90–120 seeds with nominal strength of 0.4–0.6 U/seed. The conversion factor from radioactivity mCi to air kerma strength U for $I^{125}$ is 1mCi=1.27U, where $1\;U\equiv 1\;\mu \text{Gy}\;m^{2}h^{-1}$. The test IsoCartridge initially provided by Mentor for this study had 90 seeds with nominal air kerma strength of 0.23 U/seed (designate IsoCartridge I). After intensive measurements of IsoCartridge I, a second test IsoCartridge with air kerma strength more clinically relevant was requested. It contained 69 seeds with nominal air kerma strength of 0.59 U/seed (IsoCartridge II).

### B. Isoloader dosimetry measurements

The Isoloader incorporates a radioactivity verification system using a built‐in cadmium zinc telluride (CdZnTe) solid‐state sensor with a sensitive area of $5\times 5\;{\text{mm}}^{2}$. After an IsoCartridge is mounted on the Isoloader, the preloaded seeds are pushed out of the IsoCartridge to the detection area in the Isoloader and scanned by the radiation sensor one by one. Each seed is measured at three locations with a step size of 1 mm, and the maximum reading is taken as the final result.

During measurements, the distance between the sensor and the seed is 25 mm, resulting in 0.3% coverage of the full 4π solid angle, which means that only one out of ~300 emitted photons is detected by the sensor. For example, a seed with an activity of $0.4\;\text{mCi}\;(1.5\times 10^{7}\;\text{Bq})$ would then result in the detection of about $4\times 10^{4}$ events at the sensor in a one‐second reading (assuming perfect 100% detection efficiency) with an associated statistical error of ~0.3%. However, as described later, the final measurement uncertainty is larger than 0.5%, indicating that there are other contributing factors.

To get the overall deviation, all seeds in IsoCartridge I were measured 10 times and those in IsoCartridge II 7 times using the Isoloader. More intensive measurements were performed for a few seeds in both batches in order to obtain improved characterization of the standard deviation for the seeds. The first four seeds of IsoCartridge I and II were measured 200 and 100 times, respectively. The reason that only the first few seeds were chosen was that the Isoloader does not have the option to conveniently measure randomly chosen seeds.

### C. Well chamber measurements

For comparison, 10 seeds were ejected from the two IsoCartridges and measured individually using a well‐type ionization chamber HDR‐1000Plus (Standard Imaging, Middleton, WI). The 10 ejected seeds included 4 of the intensively measured seeds of IsoCartridge I and II, and seeds 73 and 77 from IsoCartridge I. The latter 2 seeds were chosen since they showed the largest deviation in the measurements using the Isoloader.

For the well chamber measurements, each seed was vertically inserted into the seed holder and then placed in the chamber. All well chamber readings were for charges integrated over a fixed period of time as detailed below. The air kerma strength was then determined. The solid angle coverage of the well chamber is almost 100%,^(^ ^8^ ^,^ ^9^ ^)^ so good measurement reproducibility was expected.

Three seeds (seeds 2, 4, and 77 of IsoCartridge I) were measured 60 to 100 times (60 s/reading). Each seed was taken out and reinserted into the well chamber for each reading. Standard deviations of the readings for each seed turned out to be larger than expected (1.3%, 0.8%, and 1.7%).

Nonuniformity of the seeds due to capsule wall thickness, laser‐weld ends, and $I^{125}$ radioactive material coating on the silver beads may contribute significantly to the measurement uncertainty. To investigate this, each of the 10 ejected seeds was marked on one end. Then the seed was inserted into the chamber 10 to 30 times, alternately with marked end toward the top (“seed up”) and the bottom of the well chamber (“seed down”). Four readings (15 s/reading) were taken after each insertion.

## III. RESULTS

### A. Isoloader measurements

**Figure 2:**
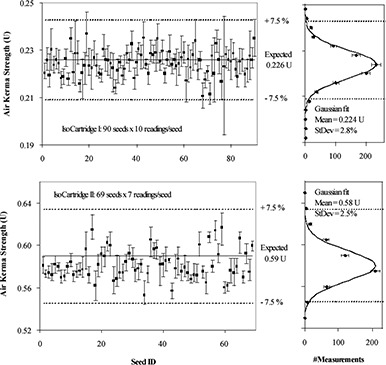
Left: Air kerma strength of individual seeds in two test IsoCartridges. All seeds were measured multiple times using the Isoloader, as noted in the plot. For each seed, the solid square (■) is the average of the readings, and the error bar represents the range of minimal to maximal reading. The thick solid line is the expected seed strength for each batch, and the two dashed lines stand for the ±7.5% variation. Right: histograms of the left plots. The error bars count statistical error, and the solid line is the Gaussian fit, with the mean value and standard deviation indicated.

The air kerma strength of individual seeds in both test IsoCartridges measured by the Isoloader is displayed in Fig. 2 (left‐hand plots). The manufacturer recommends an initial acceptance/rejection cutoff of ±7.5% for each seed when comparing the Isoloader measurement with the nominal seed strength. This cutoff is based on the combination of (1) a potential ±4.5% deviation from the nominal seed strength (in the manufacturer's calibration) and (2) a ±3.5% variation due to the measurement accuracy (95% confidence) of the Isoloader system. The manufacturer suggests that the cutoff value of ±7.5% provides sufficient measurement tolerance yet adequate quality assurance to avoid rejection of acceptable seeds. At our clinic we adopted this suggested ±7.5% criterion for “good” seeds for the initial assessment in which the seeds are evaluated through a comparison with the nominal stated seed activity rather than by the mean activity, which can be determined only after all seeds have been measured. It can be noted that this initial criterion is different from the TG40 recommendations based on the characterization of the whole batch of seeds (±3% deviation of the mean from the nominal seed strength and ±5% spread from measured mean).^(4)^ With the TG40 rejection limits, an individual seed in the initial measurements could deviate as much as ±8% from the stated strength. Considering this, we accepted the manufacturer's recommendations of ±7.5% for the initial tolerance setting.

The reading averages for each seed (the solid squares) were all within this range; thus, all the seeds were “good.” However, in the clinical situation, each seed would normally be measured only once by the Isoloader. With single measurements, the reading for each seed could be anywhere within the error bar for each seed. As a result, about eight seeds in IsoCartridge I and one seed in IsoCartridge II could have been wrongly rejected as “bad” seeds, had only a single measurement been performed on each seed with the Isoloader.

The histograms shown in the right‐hand plots of Fig. 2 were obtained using MATLAB (v6.5, The MathWorks, Natick, MA) and fitted with Gaussian functions with a bin size of 0.004 U and 0.015 U for IsoCartridges I and II, respectively. At each air kerma strength value *A*, the solid diamond is the number of readings (*N*) within the range of A±½ bin size, while the error bar indicates the statistical error in counting $\left(\sqrt{N}\right)$. The mean values of the Gaussian fit were 0.224 U and 0.58 U for IsoCartridges I and II, respectively, which was 1%~2% lower than the expected value. The standard deviation was 2.8% for IsoCartridge I and 2.5% for IsoCartridge II.

**Figure 3:**
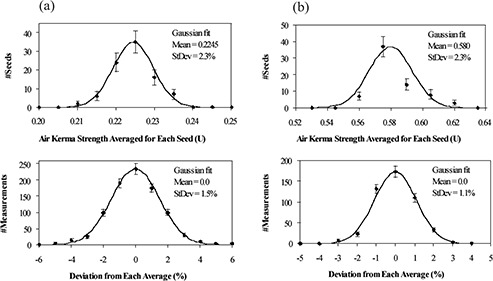
(a). Histograms derived from Isoloader measurements for IsoCartridge I. Top: Air kerma strength averaged over 10 readings for each of the 90 seeds, that is, the solid squares (■) in Fig. 2. Bin size 0.005 U. Bottom: Percentage differences between 10 readings and their average for each of the 90 seeds. Bin size 1%. Fig. 3 (b) Similar to Fig. 3(a) but for IsoCartridge II. Bin size is 0.015 U for the top plot and 1% for the bottom plot.

The deviation of the air kerma strength readings is considered to be composed of two parts: (1) seed strength fluctuation associated with manufacturing and (2) measurement uncertainty of the Isoloader. To quantify them separately, two histograms were derived for each batch. As shown in Fig. 3(a) (IsoCartridge I) and Fig. 3(b) (IsoCartridge II), the histogram at the top is the average readings of the individual seeds (e.g., 90 data points for IsoCartridge I), while the histogram at the bottom illustrates the percentage differences between the multiple readings and their average for each seed (e.g., 10 readings/seed for 90 seeds, totaling 900 data points for IsoCartridge I). Again, the histograms were fitted by Gaussian functions. The standard deviation of seed strength fluctuation was 2.3% for both IsoCartridges, while for the Isoloader measurements, it was 1.5% for IsoCartridge I and 1.1% for IsoCartridge II, which means better measurement reproducibility of the Isoloader for IsoCartridge II.

The difference in measurement reproducibility was possibly due to different nominal seed strengths of the batches at the time of measurements (0.23 U/seed for IsoCartridge I vs. 0.59 U/seed for IsoCartridge II). To assess this, we allowed the seeds in IsoCartridge II to decay for about 2 months. When their nominal strength decreased to about 0.24 U/seed, we measured IsoCartridge II again 7 times using the Isoloader. The new measurements showed a standard deviation of 2.7%, which was composed of seed strength fluctuation of 2.3% (no change) and measurement uncertainty of 1.4% (close to 1.5% when IsoCartridge I was measured with nominal strength of 0.23 U/seed).

**Figure 4:**
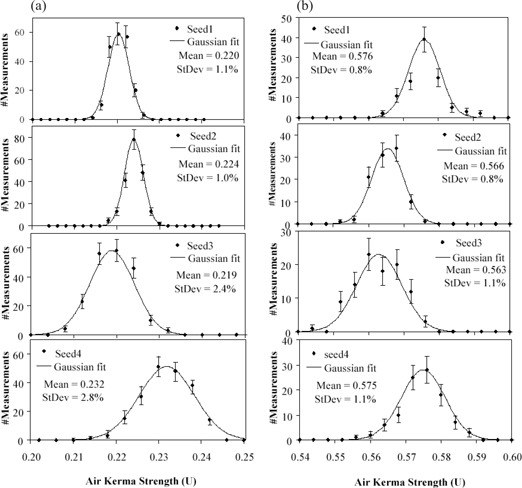
(a) Histograms of Isoloader measurements for the first four seeds in IsoCartridge I. Each seed was measured 200 times. The bin size was 0.002 U for seeds 1 and 2 and 0.004 U for seeds 3 and 4. Fig. 4 (b) Histograms of Isoloader measurements for the first four seeds in IsoCartridge II. Each seed was measured 100 times. Bin size 0.004 U.

Results from more intensive measurements of the first four seeds of both batches are shown in Figs. 4(a) and (b). The mean values of the Gaussian fits of the seeds in IsoCartridge I range from 0.219 U to 0.232 U and those in IsoCartridge II from 0.563 U to 0.576 U. Standard deviations were 1.0% to 2.8% for the seeds in IsoCartridge I and 0.8% to 1.1% for the seeds in IsoCartridge II. Two observations can be made: (1) seed strength changed from seed to seed in a batch, indicating seed manufacturing uncertainty; (2) measurement reproducibility of the Isoloader was also seed‐specific, and generally it was better for the seeds in IsoCartridge II, which was consistent with the measurements using whole batches.

### B. Well chamber measurements

**Figure 5:**
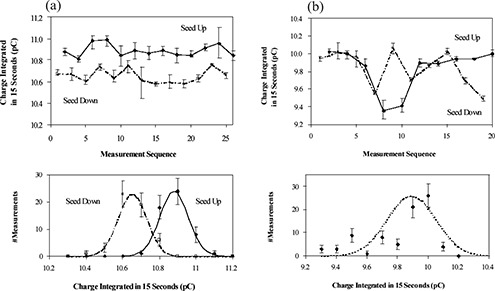
(a) Well‐type ionization chamber measurements of seed 2 in IsoCartridge I. Top: original measurements. Solid diamonds (♦) connected by a solid line correspond to “seed up” orientation; open squares (□) connected by a dashed line correspond to “seed down” orientation. For each measurement sequence, the marker (♦ or □) is the average of the four readings taken after each insertion, and the error bar represents the range of minimal to maximal reading. Bottom: histograms derived from the top plot. The error bars count statistical error, and the solid and dashed lines are the Gaussian fits for “seed up” and “seed down” orientations, respectively. Fig. 5 (b) is the same as Fig. 5(a) but for seed 77 in IsoCartridge I. Only one Gaussian fit is applied for both “seed up” and “seed down” orientations.

Each of the 10 ejected seeds was inserted into the well chamber in both “seed up” and “seed down” orientations alternately with four readings taken after each insertion. Each group of four readings is called a measurement sequence. Figures 5(a) and (b) show the results of the measurements on seeds 2 and 77 from IsoCartridge I.

The top plots of Fig. 5 show that, within each measurement sequence, in most cases the four readings agree well with one another because the seed was not disturbed; thus, the measurement uncertainty was mainly caused by statistical error. However, from one measurement sequence to another the readings do fluctuate because of repositioning and nonuniformity of the seed. The fluctuation differs from seed to seed. Comparing Figs. 5(a) and (b), we see that, for seed 2, readings are strongly related to vertical orientation of the seed (up and down), but for each orientation, the readings are fairly stable, resulting in two well‐separated Gaussian peaks in the histogram in the bottom plot. For seed 77, on the other hand, readings fluctuate more significantly but are not obviously correlated to seed vertical orientation, resulting in one broad Gaussian peak in the histogram. This may indicate different contributions to measurement uncertainty (for details see Discussion).

### C. Comparison of Isoloader and well chamber measurements

The results of the measurements of the 10 seeds using both the Isoloader and well‐type ionization chamber are summarized in Table 1. The standard deviation was seed‐specific for both Isoloader and well chamber measurements. In general, the standard deviations of the Isoloader measurements were larger than those of the well chamber measurements, except for seed 2 in both IsoCartridges, where readings were strongly related to the vertical orientation of the seed (see Fig. 5(a)). The overall measurement reproducibility was better for the seeds in IsoCartridge II, which was possibly due to the larger nominal seed strength as already explained in Section III.A.

**Table 1:** Comparison of standard deviations (SD) and mean values derived from measurements using the Isoloader (Iso) and well‐type ionization chamber (WC)

		Isoloader SD (# of meas.)	Well Chamber SD (# of meas.)	%Diff of mean (Iso‐WC) w.r.t. mean by WC
IsoCartrridge Test #1	seed 1	1.1% (200)	0.8% (10)	0.4%
	seed 2	1.0% (200)	1.3% (60)^a^	0.6%
	seed 3	2.4% (200)	1.0% (12)	2.2%
	seed 4	2.8% (200)	0.8% (100)	3.2%
	seed 73	~5%(10)	1.2% (10)	−1.0%
	seed 77	~8%(10)	1.7% (60)	8%
IsoCartrridge Test #2	seed 1	0.8% (100)	0.6% (20)	2.0%
	seed 2	0.8% (100)	1.0%(20)^a^	−0.8%
	seed 3	1.1% (100)	0.7% (10)	1.1%
	seed 4	1.1% (100)	0.7% (10)	3.1%

^a^ With two peaks in the histogram associated with seed up and down orientations. Each peak had standard deviation of 0.7% (0.5%), and the mean values of the two peaks were 2.1% (1.6%) apart for seed 2 in IsoCartridge I (IsoCartridge II).

The accuracy of the Isoloader was checked by comparing the seed strength measured using the Isoloader with that derived from well chamber readings, where the well chamber is routinely calibrated at our clinic using a calibrated I125 seed with secondary traceability to the NIST, provided by the supplier. As shown in the last column in Table 1, the mean values of Isoloader measurements were generally larger than those from the well chamber, on average, by 1%~2%.

## IV. DISCUSSION

**Figure 6:**
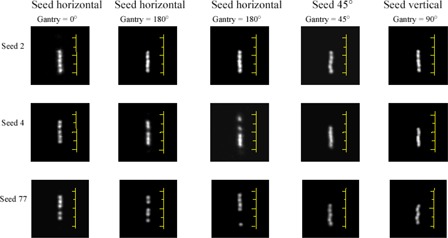
Transmission radiographs of three seeds from IsoCartridge I show the variability of five $I^{125}$‐coated silver beads inside the seed capsule. The radiographs were taken on a simulator (40 mAs, 65 kVp) at different gantry angles; thus, the seeds are at different orientations. The scale in each image represents the outer length of the seed capsule (4.5 mm). The images were enhanced using MATLAB.

The major contribution to the deviation of the Isoloader measurements was the bead variability within seed capsules. This variability was verified by taking transmission radiographs on a conventional X‐ray simulator. Several seeds were taped on a plastic tray that was attached to the gantry head of the simulator. With this setup the seeds were at different orientations as the simulator was set to different gantry angles. Figure 6 shows transmission radiographs of three of the seeds. When the seeds were placed horizontally (gantry at 0° and 180°, twice at 180°), the beads could be anywhere within the capsule and their positions changed from one radiograph to another (except for seed 2, where the beads seemed always stuck together); when the seeds were not placed horizontally (gantry at 45° and 90°), all the beads staggered to the bottom end of the capsule because of gravity.

During Isoloader measurements, the seeds were placed horizontally; thus, bead variability was potentially significant. The deviation in measurements can, therefore, be attributed mainly to the small solid angle of the detector (~0.3% of 4π) and the bead variability within seed capsules. The effect of seed nonuniformity may be insignificant because the Isoloader reads each seed at three positions and takes the maximum as the final result. In Fig. 6, it seemed that the bead variability was the largest for seed 77 and smallest for seed 2, which could explain the different magnitudes of the Isoloader measurement deviation shown in Fig. 2 (top left).

When measured with the well‐type ionization chamber, the seed was placed vertically, and the beads were staggered at the bottom end of the capsule; thus, the effect of the bead variability was reduced. Rather, the nonuniformity of the seed due to encasing as well as radioactive material coating was probably the major contribution to measurement variability. For seeds with readings strongly related to their vertical orientation (e.g., seed 2 in IsoCartridge I; see Fig. 5(a)), nonuniformity of the weld end could have played a major role because when beads staggered to the thicker weld end, smaller readings were obtained due to increased attenuation.

For the batch with nominal activity comparable to that used in clinics (IsoCartridge II), the overall standard deviation of the Isoloader measurements, 1.1%(±2% at 95% confidence), was slightly larger than that of the well chamber measurements, 0.8%. The reproducibility of the Isoloader fulfills the criterion in TG40,^(4)^ which states that “the reproducibility of the source calibrator should be better than 2%.”

Seed strength measured with the Isoloader was on average 1%~2% larger than the strength measured with the well chamber (calibration traceable to the NIST) (see Table 1), but 1%~2% smaller than the nominal ordered strength of the batch (see Fig. 2). This indicates that if all the seeds in a whole batch could be measured one by one using the well chamber, the “true” average strength of the batch would be 2%~4% smaller than ordered. This is clinically acceptable according to the criterion in TG40, which requires that the measured mean seed strength of a batch be within 3% of the expected value.

## V. CONCLUSION

An automated seed‐loading system Isoloader (Mentor Corp.) was evaluated for seed calibration in prostate brachytherapy and compared with a well‐type ionization chamber using two test IsoCartridges. Reproducibility and accuracy of I125 seed calibration using the solid‐state detector built in the Isoloader were found clinically acceptable.

## ACKNOWLEDGMENTS

The authors are thankful to Mentor Corporation for providing two test IsoCartridges and to Phil Hancock (Mentor Corp.), Dan Schmidt (Standard Imaging), and John Micka (Wisconsin University) for technical consultation.
